# Tobacco BY-2 cell-free lysate: an alternative and highly-productive plant-based in vitro translation system

**DOI:** 10.1186/1472-6750-14-37

**Published:** 2014-05-03

**Authors:** Matthias Buntru, Simon Vogel, Holger Spiegel, Stefan Schillberg

**Affiliations:** 1 Fraunhofer Institute for Molecular Biology and Applied Ecology IME, Forckenbeckstr. 6, 52074 Aachen, Germany

**Keywords:** Cell-free protein synthesis, In vitro translation, *Nicotiana tabacum* cv. BY-2, Wheat germ extract, Protein expression

## Abstract

**Background:**

Cell-free protein synthesis is a rapid and efficient method for the production of recombinant proteins. Usage of prokaryotic cell-free extracts often leads to non-functional proteins. Eukaryotic counterparts such as wheat germ extract (WGE) and rabbit reticulocyte lysate (RLL) may improve solubility and promote the correct folding of eukaryotic multi-domain proteins that are difficult to express in bacteria. However, the preparation of WGEs is complex and time-consuming, whereas RLLs suffer from low yields. Here we report the development of a novel cell-free system based on tobacco Bright Yellow 2 (BY-2) cells harvested in the exponential growth phase.

**Results:**

The highly-productive BY-2 lysate (BYL) can be prepared quickly within 4–5 h, compared to 4–5 d for WGE. The efficiency of the BYL was tested using three model proteins: enhanced yellow fluorescent protein (eYFP) and two versions of luciferase. The added mRNA was optimized by testing different 5’ and 3’ untranslated regions (UTRs). The protein yield in batch and dialysis reactions using BYL was much higher than that of a commercial Promega WGE preparation, achieving a maximum yield of 80 μg/mL of eYFP and 100 μg/mL of luciferase, compared to only 45 μg/mL of eYFP and 35 μg/mL of luciferase in WGEs. In dialysis reactions, the BYL yielded about 400 μg/mL eYFP, representing up to 50% more of the target protein than the Promega WGE, and equivalent to the amount using 5Prime WGE system.

**Conclusions:**

Due to the high yield and the short preparation time the BYL represents a remarkable improvement over current eukaryotic cell-free systems.

## Background

Cell-free protein synthesis (CFPS) systems based on crude lysates provide several advantages over *in vivo* systems and offer broad applications in protein engineering, biopharmaceutical product development and post-genomic research [1]. Crude lysates contain the necessary components for translation, protein folding, and energy metabolism, so providing them with amino acids, energy substrates, nucleotides and salts allows almost any protein encoded by a RNA template to be synthesized. In coupled transcription/translation systems supplemented additionally with an appropriate RNA polymerase DNA templates can also be used. In contrast to traditional cell-based expression methods, CFPS offers shorter process times, limited protein hydrolysis and the ability to express toxic proteins or proteins containing specific chemical groups or unnatural amino acids at defined positions [2]. Furthermore, the open nature of the system allows the reaction to be controlled and monitored directly. Although chemical synthesis allows the rapid and controlled synthesis of peptides < 40 residues in length, this is not an economically feasible method for the production of larger proteins [3].

The most widely used cell-free systems are based on *Escherichia coli* extract (ECE), wheat germ extract (WGE), rabbit reticulocytes lysate (RLL) and insect cell extract (ICE). These contain diverse cellular components and co-factors that enhance protein expression, folding and modification in different ways. Therefore, the most appropriate system will depend on the origin and the biochemical nature of the target protein. The preparation of ECE is simple and inexpensive, and generally achieves the highest protein yields, from hundreds of micrograms to milligrams per milliliter in batch reactions depending on the target protein [4,5]. In contrast, eukaryotic systems are less productive and extract preparation is more laborious, but complex proteins can be produced more efficiently and extended post-translational modifications are supported. WGE normally yields tens of micrograms to milligrams of recombinant protein per milliliter, depending on the protein and reaction format [6-8], but extract preparation takes 4–5 d, and the yield of extract from wheat seeds is low [9]. The yields of RLL systems are typically two orders of magnitude lower than WGE [10] and ICEs prepared from *Spodoptera frugiperda* can achieve yields of up to 50 μg/mL [11]. Recently two further eukaryotic systems based on CHO cells [12] and *Saccharomyces cerevisae*[13] have been described. The CHO extract yield up to 50 μg/mL active firefly luciferase, but the fermentation medium is quit expensive. In contrast the preparation of the yeast extract is inexpensive, but the system produces only low target protein levels of 8 μg/mL active firefly luciferase. The drawbacks of current cell-free systems have therefore created a demand for highly-productive eukaryotic cell-free systems that can be prepared quickly in large amounts.

Here we describe a highly-productive *in vitro* translation system derived from tobacco BY-2 cells. This type of cells was used due to the eukaryotic nature, the simple and cost-effective fermentation and the well-established genetic modification tools. The novel system was tested against commercial WGEs for the production of three model proteins: enhanced yellow fluorescent protein (eYFP), firefly luciferase (FFLuc) and *Renilla reniformis* luciferase (RRLuc). By measuring the activity of these optical reporter proteins, the rate of translation was determined without radioactive substrates. Several different 5’ and 3’ untranslated regions were also compared for their translation enhancing activity.

## Results and discussion

### Preparation of the tobacco Bright Yellow-2 lysate (BYL)

**Figure 1:**
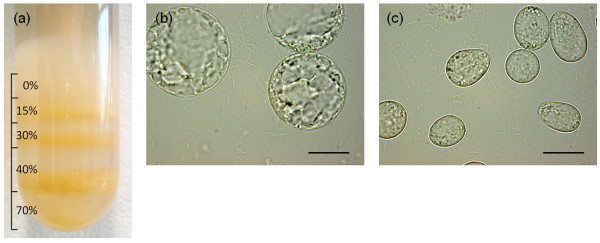
**Preparation of evacuolated tobacco BY-2 protoplasts by Percoll gradient centrifugation. (a)** Evacuolated protoplasts are concentrated at the interface between 40% and 70% Percoll. **(b)** Representative image of protoplasts before evacuolation (Scale bar = 20 μm). **(c)** Representative image of evacuolated protoplasts (Scale bar = 20 μm).

At present *in vitro* translation systems suffer from certain shortages like laborious extract preparation, low protein synthesis yields or the inability to support posttranslational modifications [14]. The aim of this study was to establish a system, allowing the rapid preparation of lysates for cell-free synthesis of satisfactory recombinant protein yields. For this purpose, we further developed a system based on BY-2 cell suspension cultures designed for the replication of plant RNA viruses [15,16]. In contrast to the original protocol using batch-cultured cells, we used BY-2 cells growing continuously in a stirred-tank fermenter at a constant packed cell volume of 20% and a doubling time of around 32 h, to ensure a reproducible supply of homogeneous cell material. We also replaced the conventional cell wall-digesting enzymes (Cellulase Onozuka RS, Pectolyase Y-23 and Macerozyme R-10) with the liquid enzymes Rohament CL, Rohament PL and Rohapect UF, originally intended for the production of fruit juice and extracts. Rohament CL comprises a cellulase concentrate, Rohament PL is a pectinase concentrate, and Rohapect UF contains an enzyme complex including specialized pectinases and arabanases. This reduced the costs for the protoplastation step more than 100-fold. However, the most important step in the lysate preparation is the removal of the lytic vacuoles (Figure 1). These contain a great part of undesirable enzymes, including proteases and ribonucleases. Lysates prepared without the evacuolation step show nearly no translation activity [15]. The vacuoles were removed by centrifugation in a Percoll gradient using a modified method based on those described by Komoda *et al.*[15] and Ishibashi *et al.*[17]. Due to its low density vacuoles can be separated from the protoplasts yielding high-density evacuolated protoplasts. The continuous Percoll layer described in the reports listed above was replaced with a stepwise density gradient, and the protoplasts were applied directly onto the Percoll-free top layer. After centrifugation, the evacuolated protoplasts concentrated at the interface between the 40% and 70% Percoll layers (Figure 1a), whereas the separated vacuoles floated on the top layer. The purity of the evacuolated protoplasts was verified by microscopy (Figure 1b,c). The yield of the evacuolation step was ~65%, estimated using a Neubauer counting chamber. The evacuolated protoplasts were washed and then disrupted by nitrogen decompression to protect labile cell components from oxidation [18]. After the removal of nuclei and non-disrupted cells, the lysate was incubated with nuclease S7 to destroy endogenous mRNA and to reduce background translation to a minimum [16]. This step leaves the integrity of the 18S and 28S ribosomal RNAs mainly unaffected and therefore increases the translation efficiency of exogenous mRNA substantially [16].

The evacuolation step is comparable to the endosperm removal step during WGE preparation, and is essential because the endosperm contains several translation inhibitors such as tritin and thionin, as well as nucleases and proteases [6,9,19]. However, the evacuolation step takes ~90 min, whereas endosperm removal requires 2–3 d because embryo particles remaining after the grinding and sieving of wheat seeds must be selected manually to eliminate those containing larger amounts of endosperm [9]. Without the preselection of embryo particles by flotation using toxic organic solvents such as carbon tetrachloride, the selection process would take even longer [9].

**Figure 2:**
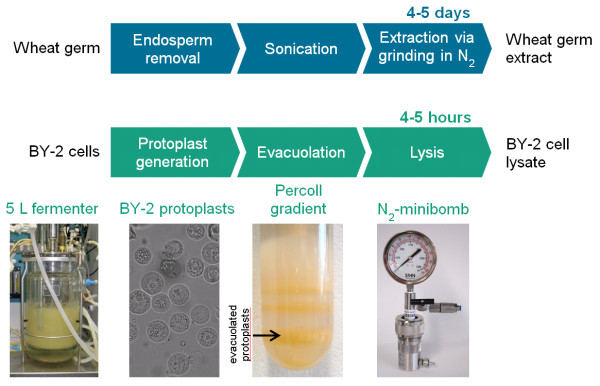
**Flowchart showing the preparation of WGE (top panel) and BYL (bottom panel).** WGE (top panel). The endosperm is removed from wheat seeds and embryo particles are washed extensively to remove translation inhibitors from the endosperm. To obtain the extract, the washed embryo particles are ground with a mortar under liquid nitrogen or in a Waring blender. BYL (bottom panel). BY-2 cells are cultivated in a 5-L fermenter, harvested in the exponential growth phase (at least 5 days post-inoculation) and converted into protoplasts. The vacuoles are separated from the protoplasts by centrifugation over a stepwise Percoll gradient. The resulting evacuolated protoplasts are lysed using the nitrogen decompression method.

The lysis of the evacuolated protoplasts takes ~30 min, which again is much faster than the equivalent process during WGE preparation, i.e. the extraction of the wheat embryos which takes 5–7 h [9]. Furthermore, the nitrogen decompression method for protoplast lysis protects labile components from oxidation, in contrast to the crushing of embryo particles in a mill/mixer or the disruption of protoplasts using a Dounce homogenizer as described in the original protocol [15,16]. Figure 2 compares the timelines for the preparation of BYL and WGE, showing that the preparation of BYL takes 4–5 h compared to 4–5 days for the WGE. A 3-L BY-2 cell suspension culture can yield ~30 mL of BYL with a final protein concentration of 14.9 ± 0.3 mg/mL [20].

### In vitro translation activity of BYL in batch reactions

The performance of BYL compared to commercial WGEs was investigated by producing the reporter proteins eYFP and luciferase in both systems and measuring fluorescence, which is much faster than standard methods and does not involve radioactive labeling. Kahn *et al.*[21] and Yukawa *et al.*[22] demonstrated that the fluorescence of GFP produced in cell-free systems was comparable with the radioactivity of ^35^S methionine incorporated during the translation process, showing that translation yield can be quantified by fluorescence measurement. Similarly, a correlation between luciferase activity and the incorporation of ^14^C leucine has been demonstrated in WGE, RLL and ECE [23,24].

**Figure 3:**
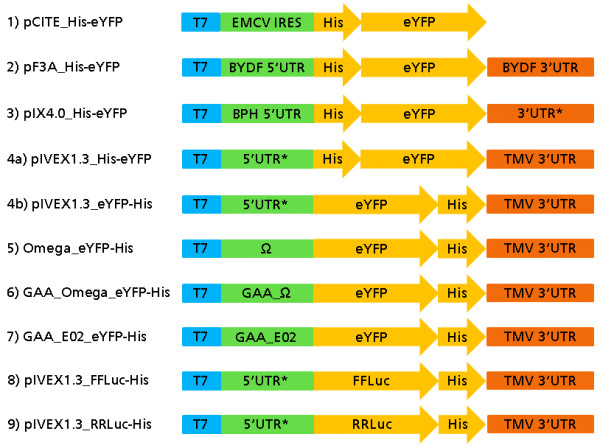
**Schematic representation of different eYFP and luciferase expression constructs with various 5’ and 3’ UTRs.** EMCV IRES, *Encephalomyocarditis virus* internal ribosomal entry site; *BYDV* 5’ UTR*, Barley yellow dwarf virus* 5’ untranslated region; BPH 5’ UTR, Baculovirus polyhedrin gene 5’ untranslated region; Ω, *Tobacco mosaic virus* 5’ leader sequence (omega); GAA_Ω, *Tobacco mosaic virus* 5’ leader sequence (omega) with GAA as first nucleotide triplet; GAA_E02, synthetic 5’ leader sequence; TMV, *Tobacco mosaic virus*; 5’ UTR*, repetition of a sequence complementary to the 18S ribosomal RNA; 3’ UTR*, trailer sequence with synthetic poly(A) signal; FFLuc, firefly luciferase; RRLuc, *Renilla reniformis* luciferase.

To maximize protein expression, we optimized the mRNA sequence introduced into the BYL translation reaction. Several mRNA structural characteristics affect translation efficiency [25] including the untranslated regions (UTRs) at the 5’ and 3’ ends of the coding sequence [26,27]. The structure of the 5’ UTR influences translational initiation, termination and mRNA stability [28]. One of the rate-limiting steps in translational initiation is the binding of the mRNA to the 43S pre-initiation complex [29]. The translational machinery is recruited by the 5’-cap or translational enhancers in the leader sequence [30-33]. To find the optimal UTRs for the BY-2 system, we constructed eight different eYFP expression constructs with various 5’ and 3’ UTRs (Figure 3). The 5’ UTR in pCITE2a contains an internal ribosomal entry site (IRES) derived from *Encephalomyocarditis virus* (EMCV), which is widely used in eukaryotic host cells and cell-free extracts [34,35]. The pF3A vector was designed for use with wheat germ extract and contains sequences from *Barley yellow dwarf virus* (BYDV) [36]. The pIX4.0 vector used with ICEs contains the 5’ UTR from a baculovirus polyhedrin gene and a synthetic 3’ UTR including a poly(A) sequence. The polyhedrin promoter achieves the highest yields in the ICE system [37-40], but because it is also compatible with the WGE and RLL systems [38] we assumed it would also be functional in the BYL. The 5’ UTR in the pIVEX1.3 vector includes an ARC-1 sequence element, which is complementary to an internal 18S rRNA segment [41] and may promote binding to the 40S ribosomal subunit [42]. This 18S RNA region is highly conserved among eukaryotes [43], so we assumed it would be functional in both the BYL and WGE systems. The *Tobacco mosaic virus* (TMV) 5’-UTR (omega sequence) is a well-known translational enhancer that is included in the vectors pIVEX_Omega_eYFP-His and pIVEX_GAA_Omega_eYFP-His [44-47]. It is functional as an enhancer not only in tobacco plants, but also in the WGE, RLL and ECE cell-free systems [44]. The TMV omega sequence was improved by adding GAA upstream of the initial GUA triplet [48]. The pIVEX_GAA_E02 construct contains a synthetic sequence which was selected by its ability to promote the formation of polysomes in WGEs [49].

**Figure 4:**
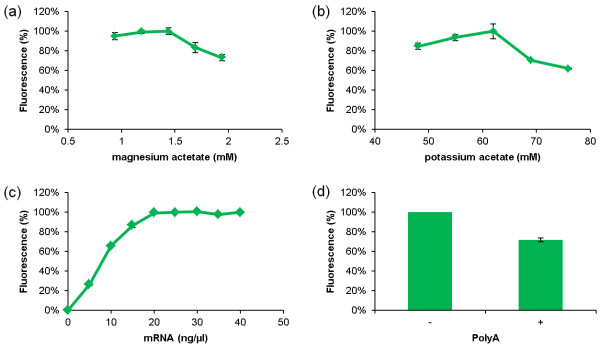
**Optimization of reaction conditions in batch mode.** Effects on eYFP yield caused by **(a)** magnesium acetate concentration, **(b)** potassium acetate concentration, **(c)** mRNA concentration, **(d)** presence (+) or absence (-) of poly(A) sequence. Translation reactions were carried out using capped GAA_Omega_eYFP-His mRNA as the template at 25°C and 500 rpm for 18 h. The relative fluorescence intensities are shown. Mean values were calculated from three independent translation experiments.

The eYFP expression constructs described above were used as templates to produce capped mRNA *in vitro* in the presence of the cap analog m^7^G[5’]ppp[5’]G. Non-incorporated nucleotides and cap analogs were removed by gel filtration, and 2 μg of the purified mRNA was translated in the BYL and WGE (Promega) systems for 18 h. The optimal reaction conditions were defined by monitoring eYFP fluorescence using capped GAA_Omega_eYFP-His mRNA as a template. The highest eYFP fluorescence was detected in the presence of 1.44 mM magnesium acetate and 61 mM potassium acetate (Figure 4a,b). Because the optimal potassium concentration is dependent on the template, each construct was evaluated in the same manner. The optimum potassium concentration ranged from 61 mM (Figure 3; constructs 1, 2, 4a, 4b, 6) to 68 mM (Figure 3; constructs 3, 5, 7). The influence of the template concentration was determined using capped GAA_Omega_eYFP-His mRNA, and saturation was observed at ~40 ng/μL (Figure 4c). Polyadenylation sequences are known to increase translational efficiency and mRNA stability [50]. So we exchanged the TMV 3’ UTR sequence in pIVEX1.3_eYFP-His and GAA_Omega_eYFP-His with the equivalent sequence in pIX4.0_His-eYFP, containing a synthetic poly(A) sequence, but this exchange actually reduced the yield of the target protein (Figure 4d). Similarly, Sawasaki *et al.*[48] found that translation in WGE does not depend on a specific 3’ UTR or a poly(A) sequence, but rather on the length of the 3’ UTR.

**Figure 5:**
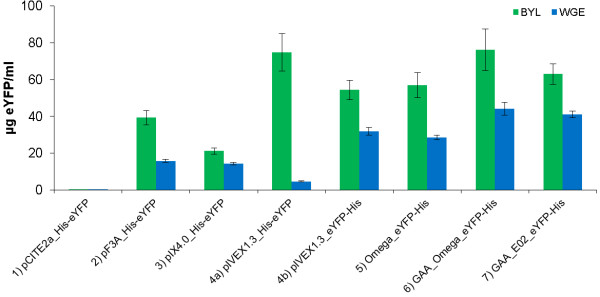
**Translation of eYFP mRNAs with various 5’ and 3’ UTRs in BYL and WGE systems in batch mode.** Reactions were carried out with 2 μg capped mRNA as the template at 25°C and 500 rpm for 18 h. The amount of fluorescent protein was calculated by comparing with an eYFP standard curve generated by measuring different eYFP concentrations in BYL translation reactions without the mRNA template. Data represent the averages and standard deviations of six independent translation experiments.

In the WGE system, we used 2.1 mM magnesium according to the manufacturer’s instructions. The optimal concentrations of potassium and mRNA were determined as described for the BYL system (data not shown). The highest fluorescence intensities were achieved at potassium concentrations of 103 mM (Figure 3, construct 2), 133 mM (constructs 1, 4a, 4b, 5, 6 and 7) and 163 mM (construct 3). As for the BYL system, 40 ng/μL of capped GAA_Omega_eYFP-His mRNA was sufficient. When the optimal concentrations were used with both systems, BYL yielded significantly more eYFP than WGE (p < 0.01) with all eight constructs (Figure 5). GAA_Omega_eYFP-His was the most productive construct in both systems, achieving maximum yields of ~78 ± 9 μg/mL eYFP in the BYL system and 44 ± 4 μg/mL eYFP in WGE batch reactions, after incubation at 25°C and 500 rpm for 18 h. Based on our findings the pCITE 5’ UTR is deemed unsuitable for both the BYL and WGE systems, even though it has previously been used for the replication of plant RNA viruses in a similar BY-2 system [16]. This may reflect the absence of specific translation factors. As expected, construct pIVEX1.3_eYFP-His worked well in both systems, probably due to the strong conservation of the 18S rRNA sequence among eukaryotes. The lysate batch variations of four different preparations tested in quadruplicates using capped GAA_Omega_eYFP-His mRNA as template was ±9% (Additional file 1).

**Figure 6:**
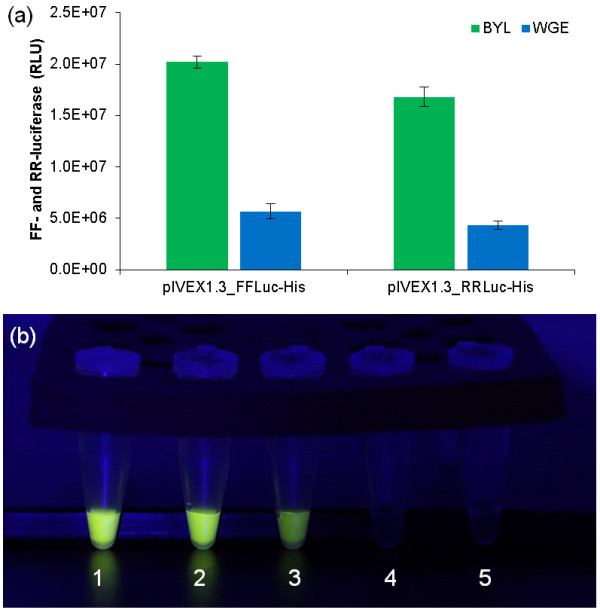
**Translation of firefly and *Renilla reniformis *mRNAs in the BYL and WGE systems.** Reactions were carried out with 2 μg capped mRNA as the template at 25°C and 500 rpm for 18 h. **(a)** Bar graphs show luciferase activity expressed as relative luciferase units (RLU). Data represent the averages and standard deviations of six independent translation experiments. **(b)** Typical result of FFLuc translation in the BYL and WGE systems, in which 1 μl of reaction mixture was combined with 50 μl luciferase assay buffer: FFLuc standard with 0.1 μg/μl (1), pIVEX1.3_FFLuc-His in BYL (2), pIVEX1.3_FFLuc-His in WGE (3), no template control in BYL (4), no template control in WGE (5).

We also compared the expression of luciferase genes from firefly (FFLuc) and *Renilla reniformis* (RRLuc) in the BYL and WGE systems, by exchanging the eYFP sequence in pIVEX1.3_eYFP-His for the corresponding luciferase genes (Figure 3). Again, we used 2 μg of capped mRNA as a template and the reactions were incubated at 25°C and 500 rpm for 18 h. The translation of both the firefly and *Renilla* luciferase RNAs was 350% higher than in the WGE, corresponding to more than ~100 μg/ml of the target protein (Figure 6a,b). These data showed that the target protein yields were similar with three different model proteins.

### In vitro translation activity of BYL in dialysis reactions

**Figure 7:**
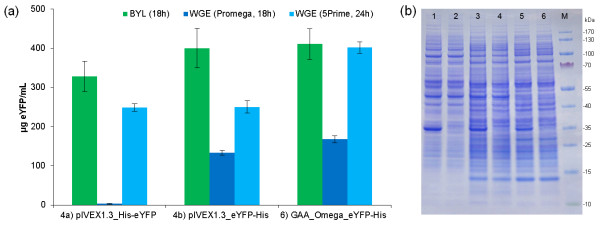
**Translation of eYFP mRNAs in BYL and WGE systems in dialysis mode.** Reactions were carried out with 3 μg capped mRNA as the template at 25°C and 900 rpm for 18 h and 24 h, respectively. The amount of fluorescent protein **(a)** was determined by comparison with an eYFP standard curve generated by measuring different eYFP concentrations in BYL translation reactions without a mRNA template. Data represent the averages and standard deviations of six independent translation experiments. **(b)** SDS-PAGE of the BYL and WGE translation reactions. In each case 10 μl of the translation reactions were loaded on a 4-12% gradient gel. Lane 1: GAA_Omega_eYFP-His in BYL; lane 2: no template control in BYL; lane 3 GAA_Omega_eYFP-His in 5Prime WGE; lane 4: no template control in 5Prime WGE; lane 5: GAA_Omega_eYFP-His in Promega WGE; lane 6: no template control in Promega WGE; M: molecular weight marker.

Although the BYL system is highly productive, sustainable *in vitro* protein synthesis can only be achieved in dialysis mode when substrates and energy components are continuously supplied across a semi-permeable membrane. Dialysis mode also dilutes potentially inhibitory reaction byproducts, which contributes to the higher protein yields. To evaluate the BYL system in dialysis mode, we used RTS Wheat Germ continuous exchange cell-free (CECF) devices (5Prime) and compared the BYL system to the two commercial WGEs from Promega and 5Prime. The reaction mixtures for the BYL and Promega WGE systems were prepared as described above for batch mode production. The feeding solutions were used in 20 fold excess and contained the same components as the reaction mixtures with the exception of lysate, mRNA, RNase inhibitor and creatine phosphokinase. The reaction mixture for the 5Prime WGE system was prepared according to the manufacturer’s instructions. We restricted the comparison to those constructs yielding the highest amounts of eYFP in the batch reactions, i.e. constructs 4a, 4b and 6 (Figure 5). We used 60 ng/μl of m_2_^7,3’-O^G[5’]ppp[5’]G capped mRNA as the template. For all three constructs, the BYL system was substantially more productive than the Promega WGE system (Figure 7a,b), producing 150% more eYFP when tested with the vectors pIVEX1.3_eYFP-His and GAA_Omega_eYFP-His. Surprisingly vector pIVEX1.3_His-eYFP was largely inactive in the Promega WGE system, but worked fine in the BYL. The BYL system was also more productive than the 5Prime WGE system, yielding ~130% more eYFP when tested with pIVEX1.3_His-eYFP and ~150% more eYFP when tested with pIVEX1.3_eYFP-His. The eYFP yield from vector GAA_Omega_eYFP-His was comparable in both systems. For construct GAA_Omega_eYFP-His in each case 10 μl of the BYL and WGE translation reactions were analysed by SDS-PAGE (Figure 7b). Notably, eYFP expression in the BYL system could be enhanced more than five-fold by switching from batch production to dialysis mode.

## Conclusions

In summary, the BYL is a promising system with high translational activity, short preparation time and high potential for scaling-up. It is likely that the efficiency of the BYL system can be improved further, e.g. by optimizing reaction conditions through a factorial design approach [51]. The BYL system is based on eukaryotic components and should therefore be suitable for the expression of eukaryotic proteins, but further studies are required to determine whether the BYL system is capable of carrying out modifications such as glycosylation and the formation of disulfide bonds. A coupled transcription/translation system based on the BYL shall also be addressed in further studies.

## Methods

### Plant material

Tobacco cells (*Nicotiana tabacum* L. cv. Bright Yellow 2, BY-2) were cultivated continuously in a 5-L fermenter (Type 100e, Applicon Biotechnology, AC Schiedam, Netherlands) with a 20% packed cell volume at 26°C in the dark in Murashige-Skoog liquid medium (Murashige and Skoog Basal Salt Mixture, Duchefa Biochemie, Haarlem, Netherlands) supplemented with 3% (w/v) sucrose, 1 mg/L thiamine-HCl, 0.2 mg/L 2,4 dichlorophenoxyacetic acid and 100 mg/L myo-inositol.

### Preparation of the BY-2 cell lysate

The preparation of lysate from evacuolated BY-2 protoplasts was carried out as described by Komoda *et al.*[15] and Gursinsky *et al.*[16] with significant modifications. Protoplasts were prepared from cells in the exponential growth phase of a continuous fermentation at a constant packed cell volume of 20% by treating the cells with 3% (v/v) Rohament CL, 2% (v/v) Rohament PL, and 0.1% (v/v) Rohapect UF (all from AB Enzymes, Darmstadt, Germany) in 0.37 mM mannitol, 5 mM CaCl_2_, 12.5 mM sodium acetate (pH 5.8) for 1.5 h. The resulting protoplasts were layered onto a discontinuous Percoll gradient containing (from bottom to top) 70% (v/v, 3 ml), 40% (v/v, 5 ml), 30% (v/v, 3 ml), 15% (v/v, 3 ml) and 0% (3 ml) Percoll (GE Healthcare, Munich, Germany) in 0.7 M mannitol, 20 mM MgCl_2_, and 5 mM PIPES-KOH (pH 7.0). After centrifugation at 12,000 g for 1 h at 25°C in a swinging bucket rotor, evacuolated protoplasts were recovered from the 40-70% (v/v) Percoll solution interface. The evacuolated protoplasts were suspended in three volumes of TR buffer (30 mM HEPES-KOH (pH 7.4), 80 mM potassium acetate, 0.5 mM magnesium acetate, 2 mM DTT) supplemented with one tablet per 50 ml of Complete EDTA-free Protease Inhibitor Mixture (Roche Diagnostics, Mannheim, Germany) and disrupted using the nitrogen decompression method in a cell disruption vessel (Parr Instrument, Frankfurt, Germany) for 30 min at 10 bar. Nuclei and non-disrupted cells were removed by centrifugation at 500 g for 10 min at 4°C. The supernatant was supplemented with 0.5 mM CaCl_2_ and treated with 75 U/ml nuclease S7 (Roche Diagnostics) for 15 min at 20°C. The lysate was supplemented with 2 mM EGTA as chelating agent for the Ca^2+^ ions to inactivate the nuclease and frozen at -80°C in 1-ml aliquots.

### DNA template preparation

Plasmid pIVEX1.3_eYFP-His was kindly provided by Dr. Stefan Kubick (Fraunhofer Institute for Biomedical Engineering, Potsdam-Golm, Germany). Plasmid pIVEX_GAA_Omega_eYFP-His was prepared by inserting annealed oligonucleotides 1 and 2 (Additional file 2) containing the T7 promoter and the TMV 5’ leader sequence (omega) into pIVEX1.3_eYFP-His using the NspI and NcoI sites. Plasmid pIVEX_GAA_E02_eYFP-His was prepared by inserting annealed oligonucleotides 3 and 4 (Additional file 2) containing the T7 promoter and the synthetic 5’ UTR E02 into pIVEX1.3_eYFP-His using the NspI and NcoI sites. Plasmids pIX4.0_His-eYFP, pIVEX1.3_His-eYFP, pF3A_His-eYFP and pCITE2a_His-eYFP were generated by amplifying the His-eYFP sequence from pIX3.0_His-eYFP (kindly provided by Dr. Stefan Kubick) using the following primers (Additional file 2): 5 and 6 for cloning into pIX4.0_eCFP (kindly provided by Dr. Stefan Kubick), 5 and 7 for cloning into pIVEX1.3_eYFP-His and pF3A (Promega, Mannheim, Germany), and 5 and 8 for cloning into pCITELuc (kindly provided by Dr. Sven-Erik Behrens, Institute of Biochemistry and Biotechnology, Halle/Saale, Germany). The PCR products were digested with BspHI and NotI, BspHI and KpnI, and BspHI and XbaI, respectively, and introduced into the NcoI and NotI sites of pIX4.0_eCFP, the NcoI and KpnI sites of pIVEX1.3_eYFP-His and pF3A, and the NcoI and XbaI sites of pCITELuc. Omega_eYFP-His was generated using a two-step PCR procedure (Sawasaki et al., 2007). In the first step, eYFP-His was amplified using pIVEX1.3_eYFP-His as a template and the two gene-specific primers 9 and 10 (Additional file 2). In the second step, the T7 promoter and omega sequence were fused to eYFP-His using primers 11 (5’ end of T7 promoter), 12 (3’ end of T7 promoter and omega sequence) and 10 as the previous step. For vector pIVEX1.3_FFLuc-His, the *FFLuc* gene was amplified by PCR using pCITELuc as the template and primers 17 and 18 (Additional file 2). The product was digested with NcoI and XhoI and reintroduced into pCITELuc in-frame with the His-tag to generate pCITE2a_FFLuc-His. Then the FFLuc-His gene was amplified using primers 17 and 19 and introduced into the NcoI and KpnI sites of pIVEX1.3_eYFP-His. The *RRLuc* gene was amplified by PCR using pSP_RRLuc (kindly provided by Dr. Sven-Erik Behrens) as the template and primers 20 and 21 (Additional file 2). The product was digested with NcoI and XhoI and inserted into pCITELuc to generate pCITE2a_RRLuc-His. The RRLuc-His sequence was then amplified using primers 20 and 21 and inserted into the NcoI and KpnI sites of pIVEX1.3_eYFP-His to generate pIVEX1.3_RRLuc-His. Plasmids pIVEX1.3_eYFP-His_Poly(A) and pIVEX_GAA_Omega_eYFP-His_Poly(A) were generated by amplifying the 3’-UTR from pIX4.0_His-eYFP by PCR using primers 22 and 23 and inserting the product into the KpnI and EcoRI sites of pIVEX1.3_eYFP-His and pIVEX_GAA_Omega_eYFP-His.

### In vitro transcription

Capped mRNA was transcribed *in vitro* in the presence of the cap analogs m^7^G[5’]ppp[5’]G or m_2_^7,3’-O^G[5’]ppp[5’]G (New England Biolabs, Ipswich, MA, USA) using the T7 High Yield RNA Synthesis Kit (New England Biolabs) and DNA templates amplified by PCR from the constructs described above using Phusion Polymerase (New England Biolabs) and the following primers (Additional file 2): 10 and 13 for pIVEX1.3_eYFP-His, pIVEX1.3_His-eYFP, pCITE2a_His-eYFP, pIVEX1.3_FFLuc-His and pIVEX1.3_RRLuc-His; 10 and 14 for pIVEX_GAA_Omega_eYFP-His, pIVEX_GAA_E02_eYFP-His and pIX4.0_His-eYFP; 10 and 11 for Omega_eYFP-His, and 15 and 16 for pF3A_His-eYFP. The RNA was purified using the DyeEx 2.0 Spin Kit (Qiagen, Hilden, Germany).

### In vitro translation

Batch reactions contained 50% (v/v) BYL, 30% (v/v) modified TR buffer (30 mM HEPES-KOH, pH 7.6, 80 mM potassium acetate, 2 mM DTT), 0.75 mM ATP, 0.1 mM GTP, 25 mM creatine phosphate, 50 μM of each amino acid, 80 μM spermine and 0.2 mg/mL creatine phosphokinase (Roche Diagnostics). The magnesium concentration was adjusted with magnesium acetate to 1.44 mM and the potassium concentration was adjusted with potassium acetate to 61 mM or 68 mM, according to the expression construct. Any magnesium and potassium already present in the BY-2 cell extracts was ignored. The batch translation reactions were carried out at 25°C and 500 rpm for 18 h in a thermomixer (Eppendorf, Hamburg, Germany). WGE (Promega) translation reactions were carried out according to the manufacturer’s instructions.

Dialysis reactions were carried out in RTS 100 Wheat Germ continuous exchange cell-free (CECF) devices (5Prime, Hamburg, Germany) using the same mixtures described for the batch reactions except that we also added 40 units of murine RNase Inhibitor (New England Biolabs). Feeding solutions contained 67.5% (v/v) modified TR buffer. For the BYL system this was 30 mM HEPES-KOH (pH 7.6), 90 mM potassium acetate, 2.1 mM magnesium acetate and 2 mM DTT, supplemented with 0.75 mM ATP, 0.1 mM GTP, 25 mM creatine phosphate, 50 μM of each amino acid and 80 μM spermine. For the Promega WGE system this was 30 mM HEPES-KOH (pH 7.6), 200 mM potassium acetate, 3.1 mM magnesium acetate and 7.4 mM DTT, supplemented with 1.2 mM ATP, 0.1 mM GTP, 10 mM creatine phosphate, 80 μM of each amino acid and 500 μM spermidine. Translation reactions in dialysis mode were carried out at 25°C and 900 rpm for 18 h in a thermomixer (Eppendorf). Translation reactions with the RTS 100 Wheat Germ CECF Kit (5Prime) were carried out according to the manufacturer’s instructions.

### Product analysis

The fluorescence signal from eYFP was quantified using a Synergy HT Multi-Mode Microplate Reader (Biotek, Bad Friedrichshall, Germany) with 485/20 nm excitation and 528/20 nm emission filters. The quantity of eYFP was determined by generating a standard curve based on different concentrations of eYFP in BYL translation reactions without a mRNA template. The eYFP standard was produced in a home-made *E. coli in vitro* translation system and purified by immobilized metal-affinity chromatography (IMAC) and size exclusion chromatography (SEC). The concentration of protein was determined using a *colorimetric assay*[20].

Firefly and *Renilla* luciferase activities were measured with the Luciferase Assay System (Promega) and a GENios Pro microplate reader (Tecan, Mainz-Kastel, Germany). Firefly luciferase purchased from Roche Diagnostics was used as a standard.

### Sodium dodecyl sulfate polyacrylamide gel electrophoresis (SDS-PAGE)

SDS-PAGE was performed using precast NuPAGE 4-12% polyacrylamide Bis-Tris gels (Life Technologies, Carlsbad, CA, USA). The gel was stained with Coomassie brilliant blue R-250. PageRuler Prestained Protein Ladder (Thermo Scientific, Waltham, MA, USA) was used as molecular weight marker.

### Availability of supporting data

The data sets supporting the results of this article are included within the article and its additional file.

## Abbreviations

BYDF: *Barley yellow dwarf virus*; BYL: BY-2 lysate; CECF: Continuous exchange cell-free; CFPS: Cell-free protein synthesis; EMCV: *Encephalomyocarditis virus*; eYFP: Enhanced yellow fluorescent protein; FFLuc: Firefly luciferase; ICE: Insect cell extract; IMAC: Immobilized metal-affinity chromatography; RLL: Rabbit reticulocyte lysate; RRLuc: *Renilla reniformis* luciferase; SEC: Size exclusion chromatography; TMV: *Tobacco mosaic virus*; UTR: Untranslated region; WGE: Wheat germ extract.

## Competing interests

We have filed a patent application relating to the use of continuous fermentation for providing cell material for preparation of the tobacco BY-2 cell-free lysate.

## Authors’ contributions

MB carried out the *in vitro* translation experiments, the product analysis and drafted the paper. SV cultured the BY2 cells and prepared the BY2 lysate. HS participated in the design of the study. SS participated in the design of the study and helped to draft the manuscript. All authors read and approved the final manuscript.

## Supplementary Material

Additional file 1 **Lysate batch variations.** The table shows the comparison of translation activities of four lots of BYL preparations. Translation reactions were carried out using capped GAA_Omega_eYFP-His mRNA as the template at 25°C and 500 rpm for 16 h, and fluorescence intensity was measured. Average and standard deviation were calculated from four translation experiments. Click here for file

Additional file 2 **Oligonucleotides used in this study. **The table shows the oligonucleotides used for the cloning and preparation of DNA templates for *in vitro* transcription. Click here for file

## References

[B1] Swartz JR Transforming biochemical engineering with cell-free biology AIChE Journal 2012 58 5 13 10.1002/aic.13701

[B2] White ER Reed TM Ma Z Hartman MCT Replacing amino acids in translation: Expanding chemical diversity with non-natural variants Methods 2013 60 70 74 10.1016/j.ymeth.2012.03.015 23718982

[B3] Nilsson BL Soellner MB Raines RT Chemical synthesis of proteins Annu Rev Biophys Biomol Struct 2005 34 91 118 10.1146/annurev.biophys.34.040204.144700 15869385 PMC2845543

[B4] Kim HC Kim TW Kim DM Prolonged production of proteins in a cell-free protein synthesis system using polymeric carbohydrates as an energy source Process Biochem 2011 46 6 1366 1369 10.1016/j.procbio.2011.03.008

[B5] Zawada JF Yin G Steiner AR Yang J Naresh A Roy SM Gold DS Heinsohn HG Murray CJ Microscale to manufacturing scale-up of cell-free cytokine production–a new approach for shortening protein production development timelines Biotechnol Bioeng 2011 108 7 1570 1578 10.1002/bit.23103 21337337 PMC3128707

[B6] Madin K Sawasaki T Ogasawara T Endo Y A highly efficient and robust cell-free protein synthesis system prepared from wheat embryos: plants apparently contain a suicide system directed at ribosomes Proc Natl Acad Sci USA 2000 97 2 559 564 10.1073/pnas.97.2.559 10639118 PMC15369

[B7] Slater MR Hurst R Pferdehirt B White D Niles A Betz N Schenborn E Expression of Soluble Native Human Proteins in Cell-Free Extracts Promega Notes 2005 91 22 25

[B8] Zhao KQ Hurst R Slater MR Bulleit RF Functional protein expression from a DNA based wheat germ cell-free system J Struct Funct Genomics 2007 8 4 199 208 10.1007/s10969-007-9035-2 18034374

[B9] Takai K Sawasaki T Endo Y Practical cell-free protein synthesis system using purified wheat embryos Nature protocols 2010 5 2 227 238 10.1038/nprot.2009.207 20134421

[B10] Jackson M Boutell J Cooley N He M Cell-free protein synthesis for proteomics Briefings in functional genomics and proteomics 2004 2 308 319 10.1093/bfgp/2.4.308 15163366

[B11] Ezure T Suzuki T Shikata M Ito M Ando E A cell-free protein synthesis system from insect cells Methods Mol Biol 2010 607 31 42 10.1007/978-1-60327-331-2_4 20204846

[B12] Brodel AK Sonnabend A Kubick S Cell-free protein expression based on extracts from CHO cells Biotechnol Bioeng 2014 111 1 25 36 10.1002/bit.25013 24018795

[B13] Hodgman CE Jewett MC Optimized extract preparation methods and reaction conditions for improved yeast cell-free protein synthesis Biotechnol Bioeng 2013 110 10 2643 2654 10.1002/bit.24942 23832321

[B14] Carlson ED Gan R Hodgman CE Jewett MC Cell-free protein synthesis: applications come of age Biotechnol Adv 2012 30 5 1185 1194 10.1016/j.biotechadv.2011.09.016 22008973 PMC4038126

[B15] Komoda K Naito S Ishikawa M Replication of plant RNA virus genomes in a cell-free extract of evacuolated plant protoplasts Proc Natl Acad Sci USA 2004 101 7 1863 1867 10.1073/pnas.0307131101 14769932 PMC357018

[B16] Gursinsky T Schulz B Behrens SE Replication of Tomato bushy stunt virus RNA in a plant in vitro system Virology 2009 390 2 250 260 10.1016/j.virol.2009.05.009 19520410

[B17] Ishibashi K Komoda K Ishikawa M In vitro translation and replication of tobamovirus RNA in a cell-free extract of evacuolated tobacco BY-2 protoplasts 2006 Berlin: Springer

[B18] Loewus MW Loewus F The Isolation and Characterization of d-Glucose 6-Phosphate Cycloaldolase (NAD-Dependent) from Acer pseudoplatanus L. Cell Cultures: Its Occurrence in Plants Plant Physiol 1971 48 3 255 260 10.1104/pp.48.3.255 16657775 PMC396843

[B19] Endo Y Sawasaki T High-throughput, genome-scale protein production method based on the wheat germ cell-free expression system Biotechnol Adv 2003 21 8 695 713 10.1016/S0734-9750(03)00105-8 14563476

[B20] Bradford MM A rapid and sensitive method for the quantitation of microgram quantities of protein utilizing the principle of protein-dye binding Anal Biochem 1976 72 248 254 10.1016/0003-2697(76)90527-3 942051

[B21] Kahn TW Beachy RN Falk MM Cell-free expression of a GFP fusion protein allows quantitation in vitro and in vivo Curr Biol 1997 7 4 R207 208 10.1016/S0960-9822(06)00100-X 9162490

[B22] Yukawa M Kuroda H Sugiura M A new in vitro translation system for non-radioactive assay from tobacco chloroplasts: effect of pre-mRNA processing on translation in vitro Plant J 2007 49 2 367 376 10.1111/j.1365-313X.2006.02948.x 17156414

[B23] Kolb VA Makeyev EV Spirin AS Folding of firefly luciferase during translation in a cell-free system The EMBO journal 1994 13 15 3631 3637 8062837 10.1002/j.1460-2075.1994.tb06670.x PMC395268

[B24] Kolb VA Makeyev EV Spirin AS Co-translational folding of an eukaryotic multidomain protein in a prokaryotic translation system J Biol Chem 2000 275 22 16597 16601 10.1074/jbc.M002030200 10748063

[B25] Kozak M Regulation of translation via mRNA structure in prokaryotes and eukaryotes Gene 2005 361 13 37 16213112 10.1016/j.gene.2005.06.037

[B26] Fan Q Treder K Miller WA Untranslated regions of diverse plant viral RNAs vary greatly in translation enhancement efficiency BMC Biotechnol 2012 12 22 10.1186/1472-6750-12-22 22559081 PMC3416697

[B27] Sonenberg N mRNA translation: influence of the 5' and 3' untranslated regions Curr Opin Genet Dev 1994 4 2 310 315 10.1016/S0959-437X(05)80059-0 8032210

[B28] Sachs AB Sarnow P Hentze MW Starting at the beginning, middle, and end: translation initiation in eukaryotes Cell 1997 89 6 831 838 10.1016/S0092-8674(00)80268-8 9200601

[B29] Herrshey JWB Merrick WC The pathway and mechanism of initiation of protein synthesis 2000 New York: Cold Spring Harbor

[B30] Dreher TW Miller WA Translational control in positive strand RNA plant viruses Virology 2006 344 1 185 197 10.1016/j.virol.2005.09.031 16364749 PMC1847782

[B31] Kneller EL Rakotondrafara AM Miller WA Cap-independent translation of plant viral RNAs Virus Res 2006 119 1 63 75 10.1016/j.virusres.2005.10.010 16360925 PMC1880899

[B32] Nicholson BL White KA 3' Cap-independent translation enhancers of positive-strand RNA plant viruses Curr Opin Virol 2011 1 5 373 380 10.1016/j.coviro.2011.10.002 22440838

[B33] Walsh D Mohr I Viral subversion of the host protein synthesis machinery Nat Rev Microbiol 2011 9 12 860 875 10.1038/nrmicro2655 22002165 PMC7097311

[B34] Elroy-Stein O Fuerst TR Moss B Cap-independent translation of mRNA conferred by encephalomyocarditis virus 5' sequence improves the performance of the vaccinia virus/bacteriophage T7 hybrid expression system Proc Natl Acad Sci USA 1989 86 16 6126 6130 10.1073/pnas.86.16.6126 2548200 PMC297788

[B35] Parks GD Duke GM Palmenberg AC Encephalomyocarditis virus 3C protease: efficient cell-free expression from clones which link viral 5' noncoding sequences to the P3 region J Virol 1986 60 2 376 384 3021972 10.1128/jvi.60.2.376-384.1986 PMC288903

[B36] Wang S Miller WA A sequence located 4.5 to 5 kilobases from the 5' end of the barley yellow dwarf virus (PAV) genome strongly stimulates translation of uncapped mRNA J Biol Chem 1995 270 22 13446 13452 10.1074/jbc.270.22.13446 7768947

[B37] Ding H Griesel C Nimtz M Conradt HS Weich HA Jager V Molecular cloning, expression, purification, and characterization of soluble full-length, human interleukin-3 with a baculovirus-insect cell expression system Protein Expr Purif 2003 31 1 34 41 10.1016/S1046-5928(03)00138-4 12963338

[B38] Suzuki T Ito M Ezure T Kobayashi S Shikata M Tanimizu K Nishimura O Performance of expression vector, pTD1, in insect cell-free translation system J Biosci Bioeng 2006 102 1 69 71 10.1263/jbb.102.69 16952840

[B39] Wickham TJ Davis T Granados RR Shuler ML Wood HA Screening of insect cell lines for the production of recombinant proteins and infectious virus in the baculovirus expression system Biotechnol Prog 1992 8 5 391 396 10.1021/bp00017a003 1369220

[B40] Yamaji H Hirakawa D Tagai S Fukuda H Production of protein kinase C-delta by the baculovirus-insect cell system in serum-supplemented and serum-free media J Biosci Bioeng 2003 95 2 185 187 16233389 10.1016/s1389-1723(03)80126-3

[B41] Akbergenov RZ Zhanybekova SS Kryldakov RV Zhigailov A Polimbetova NS Hohn T Iskakov BK ARC-1, a sequence element complementary to an internal 18S rRNA segment, enhances translation efficiency in plants when present in the leader or intercistronic region of mRNAs Nucleic Acids Res 2004 32 1 239 247 10.1093/nar/gkh176 14718549 PMC373286

[B42] Matveeva OV Shabalina SA Intermolecular mRNA-rRNA hybridization and the distribution of potential interaction regions in murine 18S rRNA Nucleic Acids Res 1993 21 4 1007 1011 10.1093/nar/21.4.1007 8451167 PMC309236

[B43] Sarge KD Maxwell ES Evidence for a Competitive-Displacement Model for the initiation of protein synthesis involving the intermolecular hybridization of 5 S rRNA, 18 S rRNA and mRNA FEBS Lett 1991 294 3 234 238 10.1016/0014-5793(91)81437-D 1756865

[B44] Gallie DR Sleat DE Watts JW Turner PC Wilson TM The 5'-leader sequence of tobacco mosaic virus RNA enhances the expression of foreign gene transcripts in vitro and in vivo Nucleic Acids Res 1987 15 8 3257 3273 10.1093/nar/15.8.3257 3575095 PMC340728

[B45] Zaccomer B Haenni AL Macaya G The remarkable variety of plant RNA virus genomes J Gen Virol 1995 76 Pt 2 231 247 7844547 10.1099/0022-1317-76-2-231

[B46] Gallie DR Walbot V Identification of the motifs within the tobacco mosaic virus 5'-leader responsible for enhancing translation Nucleic Acids Res 1992 20 17 4631 4638 10.1093/nar/20.17.4631 1408765 PMC334194

[B47] Gallie DR Translational control of cellular and viral mRNAs Plant Mol Biol 1996 32 1–2 145 158 8980478 10.1007/BF00039381

[B48] Sawasaki T Ogasawara T Morishita R Endo Y A cell-free protein synthesis system for high-throughput proteomics Proc Natl Acad Sci USA 2002 99 23 14652 14657 10.1073/pnas.232580399 12409616 PMC137474

[B49] Kamura N Sawasaki T Kasahara Y Takai K Endo Y Selection of 5'-untranslated sequences that enhance initiation of translation in a cell-free protein synthesis system from wheat embryos Bioorg Med Chem Lett 2005 15 24 5402 5406 10.1016/j.bmcl.2005.09.013 16213724

[B50] Kozak M How strong is the case for regulation of the initiation step of translation by elements at the 3' end of eukaryotic mRNAs? Gene 2004 343 1 41 54 10.1016/j.gene.2004.08.011 15563830

[B51] Vasilev N Gromping U Lipperts A Raven N Fischer R Schillberg S Optimization of BY-2 cell suspension culture medium for the production of a human antibody using a combination of fractional factorial designs and the response surface method Plant Biotechnol J 2013 11 7 867 874 10.1111/pbi.12079 23721307

