# Supplementary material for: Tobacco BY-2 cell-free lysate: an alternative and highly-productive plant-based in vitro translation system
Source: BMC Biotechnol. 2014 May 3;14:37. doi: 10.1186/1472-6750-14-37 (PMC4101825; doi:10.1186/1472-6750-14-37)
Supplement: Additional file 1 — Lysate batch variations. The table shows the comparison of translation activities of four lots of BYL preparations. Translation reactions were carried out using capped GAA_Omega_eYFP-His mRNA as the template at 25°C and 500 rpm for 16 h, and fluorescence intensity was measured. Average and standard deviation were calculated from four translation experiments. [file 1472-6750-14-37-S1.docx]

# Additional files

Additional file 1 Lysate batch variations.

The table shows the comparison of translation activities of four lots of BYL preparations. Translation reactions were carried out using capped GAA_Omega_eYFP-His mRNA as the template at 25°C and 500 rpm for 16 h, and fluorescence intensity was measured. Average and standard deviation were calculated from four translation experiments.

| Lysate | Relative fluorescence units | | | | | |
| --- | --- | --- | --- | --- | --- | --- |
|  | **1** | **2** | **3** | **4** | **Average** | **SD** |
| lot 1 | 27998 | 27845 | 28778 | 28778 | 28350 | 432 |
| lot 2 | 29591 | 30303 | 30532 | 30241 | 30167 | 350 |
| lot 3 | 25210 | 25560 | 25912 | 25717 | 25600 | 257 |
| lot 4 | 32089 | 31868 | 32961 | 32689 | 32402 | 441 |
|  |  |  |  |  | 29130 | ±2493 |
