# Supplementary material for: Tobacco BY-2 cell-free lysate: an alternative and highly-productive plant-based in vitro translation system
Source: BMC Biotechnol. 2014 May 3;14:37. doi: 10.1186/1472-6750-14-37 (PMC4101825; doi:10.1186/1472-6750-14-37)
Supplement: Additional file 2 — Oligonucleotides used in this study. The table shows the oligonucleotides used for the cloning and preparation of DNA templates for in vitro transcription. [file 1472-6750-14-37-S2.docx]

Additional file 2 Oligonucleotides used in this study.

The table shows the oligonucleotides used for the cloning and preparation of DNA templates for *in vitro* transcription.

| Number | Sequence (5’ → 3’) |
| --- | --- |
| 1 | TAATACGACTCACTATAGAAAGAGTATTTTTACAACAATTACCAACAACAACAACAAACAACAACAACATTACATTTTACATTCTACAACTAC |
| 2 | CATGGTAGTTGTAGAATGTAAAATGTAATGTTGTTGTTGTTTGTTGTTGTTGTTGGTAATTGTTGTAAAAATACTCTTTCTATAGTGAGTCGTATTACATG |
| 3 | TAATACGACTCACTATAGAAAGACTCACCTATCTCTCTACACAAAACATTTCCCTACATACAACTTTCAACTTCCTATTC |
| 4 | CATGGAATAGGAAGTTGAAAGTTGTATGTAGGGAAATGTTTTGTGTAGAGAGATAGGTGAGTCTTTCTATAGTGAGTCGTATTACATG |
| 5 | GGTAGTTCATGAAACATCATCACCATCACCAC |
| 6 | GATCATGCGGCCGCTTATTACTTGTACAGCTCGTCC |
| 7 | GATCATGGTACCTTATTACTTGTACAGCTCGTCC |
| 8 | GATCATTCTAGATTATTACTTGTACAGCTCGTCC |
| 9 | CCACCCACCACCACCAATGGTGAGCAAGGGCGAGGA |
| 10 | AAGGCGATTAAGTTGGGTAACG |
| 11 | GCGTAGCTAATACGACTCACT |
| 12 | CGACTCACTATAGTATTTTTACAACAATTACCAACAACAACAACAAACAACAACAACATTACATTTTACATTCTACAACTACCACCCACCACCACCAATG |
| 13 | CTTCCGGCTCGTATGTTGTG |
| 14 | TCGCCACCTCTGACTTGAGC |
| 15 | GCCTTTTTGCGTTTCTACAAACTC |
| 16 | CGCACATTTCCCCGAAAAGTG |
| 17 | GATCATCCATGGGATCCTTCAACTTC |
| 18 | CCTAGTCTCGAGCAATTTGGACTTTCCGCC |
| 19 | CCTAGTGGTACCGATCGGAGATCTCAGTGGTG |
| 20 | GTACGACCATGGAAGACGCCAAAAAC |
| 21 | CCATGTCTCGAGCACGGCGATCTTTCCGC |
